# Sensory evaluation of poultry meat: A comparative survey of results from normal sighted and blind people

**DOI:** 10.1371/journal.pone.0210722

**Published:** 2019-01-30

**Authors:** Krzysztof Damaziak, Adrian Stelmasiak, Julia Riedel, Żaneta Zdanowska-Sąsiadek, Mateusz Bucław, Dariusz Gozdowski, Monika Michalczuk

**Affiliations:** 1 Department of Animal Breeding and Production, Poultry Breeding Division, University of Life Sciences, Warsaw, Poland; 2 Department of Technique and Food Development, Division of Engineering in Nutrition, University of Life Sciences, Warsaw, Poland; 3 Department of Animal Improvement, Institute of Genetics and Animal Breeding, Polish Academy of Sciences, Magdalenka, Poland; 4 Department of Poultry and Ornamental Bird Breeding, Faculty of Biotechnology and Animal Husbandry, West Pomeranian University of Technology, Szczecin, Poland; 5 Department of Experimental Design and Bioinformatics, University of Life Sciences, Warsaw, Poland; Tokat Gaziosmanpasa University, TURKEY

## Abstract

Visual assessment is one of the key criteria in the sensory evaluation of foods. The appearance of food products may affect their perception by other senses, sometimes giving a false picture of their quality. A true assessment of such sensory attributes as aroma, taste, tenderness, and juiciness, which are components of the overall liking of food, without the use of instrumental methods is feasible only by blind people. We have advanced a hypothesis that blindness may modify the impressions perceived through other senses used in food evaluation. To confirm this hypothesis, a sensory testing of cooked breast and leg meat from various poultry species was conducted by normal sighted and blind panelists aged from 18 to 26 years. It has been demonstrated that the lack of sight is compensated by other senses, the intensified perception of which enables a more precise sensory evaluation of food in terms of such parameters as the aroma, tenderness and juiciness. Thus, blind people can be recommended as panelists evaluating the sensory profile of food products. Scores given by the sensory panel allowed the conclusion that the most desirable poultry meat was BM of broiler chicken and capon, followed by Guinea fowl. Lower scores were given by the panelists to meat of water fowl (goose, duck), whereas the lowest ones were assigned to cooked ostrich meat.

## Introduction

Until recently, one of the most important criteria for food choice was its price. However, increased nutritional awareness by consumers, especially in developing countries, has made quality a new driving force behind purchase decisions. The concept of quality encompasses a multitude of headwords, the most common of which being: methods of food acquisition/production, health safety of food, its nutritive value, and sensory properties. The latter factors–despite being regarded as the most important quality traits of food products–are the most difficult to measure and the most subjective. Of course, there are several analytical and instrumental methods employed for food product assessment, including microbiological procedures, instrumental analyses of tenderness, springiness, color, taste, and other traits of the widely understood food quality. Unfortunately, many food products differing in sensory attributes may achieve identical scores when assessed with these traditional methods. Therefore, sensory evaluation based on direct impressions remains a reference method and an invaluable measurement tool in food quality assessment [1].

In simple words, sensory evaluation may be described as a scientific discipline which measures the properties of a product with the use of human senses [2]. A normal person uses five senses (sight, smell, hearing, taste, and touch) and engages all of these in different ways and with various intensities to establish the quality of food products [3]. Taste is the strongest creator of human sensations concerning food, especially given that humans may discriminate 5 types (sweet, sour, salty, bitter, and umami) at up to 30 intensity levels [4]. However, it has been demonstrated several times that it is sight which is the driving force shaping our assessment of food products and which often modifies the impressions perceived through other senses [5,6,7]. It was as early as in 1936 that Apicius coined the phrase “*We eat first with our eyes*” [8]. Ever since, successive developments in medical knowledge in the field of neurology, anatomy, and physiology have proved this saying right [9,10,11,12]. Today, an extension of knowledge concerning human perception of food quality through senses other than taste seems to be more important than ever. This results from the rapid advance in various culinary techniques and technologies, advertisements for food products as well as the increased popularity of many Celebrity ‘chefs’ observed in the last 50 years. Owing to the use of specialized TV, graphical tools, and photoshopping or using eye-catching kitchenware, the media present foods in a completely unrealistic light [13]. Food depicted in this way, regardless of its quality, arouses strong desirability in the viewer. Increasingly often this way of food advertising is pejoratively called ‘gastroporn’ or ‘food porn’ [5]. Negative outcomes of such marketing include, among other things, the growing incidence of obesity, especially in children and adolescents, and problems with hypertension and other diet-related diseases [14].

The awareness that food does not always taste as good as it looks encourages the search for an explicit answer to the question of whether sight may indeed modify the perception of impressions offered by other senses in such a way that the same food product will be differently evaluated by various persons. The key to this answer is finding an appropriate group of panelists who, when evaluating a food product, would not rely on their sight. Obviously, the simplest way would be to blindfold the eyes of panelists, but we still need to bear in mind the so-called “visual memory” [15]. Consequently, testing with the same persons–once blindfolded and once with uncovered eyes–could give false results. The only group of panelists without a “visual memory” and who pay no attention to the appearance of a food product during assessment are persons completely blind from birth. A sensory assessment conducted by the blind may also be less subjective for other reasons. One of these is the fact that “blindness may lead to a compensatory sharpening of the other senses” [16]. In addition, the blind-from-birth may have various perception capabilities, which to some extent compensate for their blindness [17,18].

Poultry meat was selected for our study out of the immense variety of food products based on the growing contribution of poultry products in diets of both developing and developed populations [19]. Unlike beef and pork, poultry meat is additionally not subject to religious restrictions [20]. Consumers perceive it as healthy and with a high nutritional value, owing to which it is regularly consumed by most consumers, except for those who do not eat meat. The high level of consumption of poultry products also results from their relatively low price, ease to prepare, and flavor. Compared to meat of other livestock, another factor for poultry meat is that it is available in quite a wide range of forms. Starting from the diversity of commonly available species of domesticated fowl, consumers may choose between breast muscles and leg muscles as well as between white breast meat of the gallinaceous birds (e.g., chicken, turkey) and the red meat including leg muscles of all species and breast muscles of water fowl (duck, goose).

To date, no meat sensory quality tests have been conducted with the participation of blind persons, thus the objective of this study was to verify whether blind persons may evaluate the same food products differently than normal sighted consumers, and if so how big the difference can be. Thus, we have constructed a hypothesis, where blind persons compared with sighted persons would use the remaining senses with higher intensity. We suspect, that in the situations where evaluation of a given product will differ between both panelist groups, the one declared by blind persons is more reliable—as it is not burdened with the overall appearance, and thus it will solely concern sensory properties of a product, and not its visual presentation, which can be easily falsified to manipulate the purchasing intention of a consumer. In addition, we expect that thanks to the method used (participation of blind people) the study results will provide will provide an answer to the question of which of the offered range of poultry meat is the best in terms of the evaluated attributes. The study will also enable the demonstration of whether consumer preferences concerning exclusively sensory impressions are consistent with purchasing preferences on the market which are connected with economic factors such as price or convenience of preparation for consumption.

## Materials and methods

All sighted participants signed informed consent prior to taking part in the study. All blind participants gave an oral consent, which was signed by a formal guardian–Director of the Elżbieta Róża Czacka Society for the Care of the Blind in Laski (PL). Data obtained within the study have been protected in accordance with the Security Policy assumed at the SGGW- Regulation of His Magnificence Rector No. 88/2013 of 3 December 2013. The study was approved by the Warsaw University of Life Sciences Ethics Committee for Scientific Research with Participation of People.

### Panelists

Two groups of panelists participated in the study. The first group was composed of 132 normal sighted persons, including 83 men and 49 women aged from 19 to 23 years (SD; M = 1.4, W = 1.6). They were recruited at random from among students of Warsaw University of Life Sciences (PL) via e-mail or leaflets. Among all those who volunteered, only those were selected who declared that their senses of taste and smell were not debilitated as a result of disease, who declared they consumed poultry meat at least once a week, and who were not allergic to any component of food products of animal origin. The second group was composed of 103 persons completely blind from birth, including 58 men and 45 women aged from 18 to 26 years (SD; M = 2.6, W = 3.0). They were selected from among charges of the Elżbieta Róża Czacka Society for the Care of the Blind in Laski (PL), according to the same criteria as the sighted panelists. In addition, only those persons were selected whose blindness was a congenital defect not correlated with any other sensory disorders. All blind persons spent 5 days a week in the Elżbieta Róża Czacka Society where they were nourished (breakfast, dinner, supper) in the canteen with a balanced diet consisting of meat (including poultry), vegetables, dairy products, bread and fruit. All blind persons returned home for two days a week, where their nutrition was largely determined by their caregivers. Thus, the diet of the blind people was considerably less varied in comparison to sighted people, who determined what and when to eat on their own. None of the panelists declared food neophobia. One of the blind persons, who demonstrated fear of consuming an unknown type of meat was released from participation in the study. The panelists were not identified and grouped in terms of eating habits and the frequency of consuming poultry meat. All panelists were of Polish nationality–i.e. Slavs. During the entire experiment, statistical analyses were performed for the scores given only by those persons who were involved in both sessions of assessment (see point 2.3). Experimental procedures and study objectives were explained in detail to all participants and also to their carers, especially the staff of The Society for the Care of the Blind. This was performed during a training organized before the experiment, so that they could join the study being fully aware of its goals. The panelists were trained according to Polish guidelines [21,22]. They were informed that the object of the study was poultry meat, but did not know the species of birds or the type of meat (muscle) being evaluated.

### Meat characteristics

Before the study, experimental material was collected in the form of whole carcasses of the following birds: broiler chickens, turkeys, barbarie ducks (Muscovy), capons (surgically castrated and specially fattened young rooster.), guinea fowls, goose, as well as leg muscles from ostrich. Fresh carcasses were purchased in a supermarket belonging to a popular retail network, whereas ostrich meat was acquired directly from the producer (S1 Fig). The carcasses and ostrich meat were packed in VSP packages (99% vacuum is equal to 1.3 KPa) (EDESSA–Spain), and then frozen at a temperature of −22°C until analyzed.

### Meat preparation for sensory study

Two sessions of assessment were conducted with each group of panelists, at 7 d intervals: the first one concerned breast muscles (*Pectoralis superficialis;* BM), and the second concerned leg muscles (*Ilio tibiali;* LM) (S1 Fig). Because ostrich meat intended for culinary purposes includes mainly leg muscles, 6 types of meat were assessed in the first session and 7 types of meat in the second session. Meat was defrosted at a temperature of 0±2°C in a KÜPERSBUCH cold store for 24 h. Afterwards, whole muscles were subjected to heat treatment with the Sous–vide method in a circulator (Lotus, 2016, Treviso, Italy). Breast muscles without skin and internal fat were formatted and unified to the weight of 400±15 g. Next, they were vacuum-packed using an EDDESA apparatus (VAC-20SL 2A, 2014, Barcelona, Spain) into PP polypropylene bags and immersed in a water bath at 75°C in a circulator under cover (S1 Fig). The heat treatment lasted 40–75 min until the temperature of 72°C was reached in the geometric center of the product. After completed heat treatment, meat samples were collected out from packages. Meat juice for cooking yield (CL) determination was decanted to gastro norm (GN) containers, individually for each species. The heat-treated muscles were cut crosswise into representative samples weighing 15±5 g and placed in their own juice (CL) in GN containers under cover. The containers with meat were placed into bain-maries (water temperature of 60°C). The samples were served immediately after being collected out from the bain-maries (S2 Fig).

### Consumer testing

Consumer testing was conducted at the laboratory of the Division of Engineering in Nutrition, Faculty of Human Nutrition and Consumer Sciences of Warsaw University of Life Sciences, equipped with individual stations. Throughout the tests, a temperature of 22°C (±0.5°C) was maintained in the whole laboratory by a controlled air-conditioning system. Incandescent lighting reached ca. 500 lx [23]. Five persons simultaneously conducted the testing; however, each blind person was accompanied by their carer.

Directly before the testing, each station was equipped with a white plastic tray onto which 6 white carton trays and white plastic forks were placed in the first session and 7 white cartoon trays and white plastic forks in the second session (Dart Container, Co). The trays were marked with a three-digit code, with the last digit indicating a specific sample: 1.–broiler chicken, 2.–turkey, 3.–barbarie duck, 4.–capon, 5.–guinea fowl, 6.–goose, and 7.–ostrich. The samples were always served for the assessment in the same order. After assessment of each sample, the panelists neutralized its taste with a sip of bitter, slightly cooled tea and a bite from a wheat roll. The sighted persons were given pens and evaluation sheets for systematic completion during testing. The blind people received the same sheets which were completed by their carers, together with sheets in Braille (Mountbatten Brailler Whisperer Plus) (S3 Fig). Each sheet contained brief instructions on how it had to be completed and a short definition of each attribute. The following attributes were assessed in each sample: meat color (only sighted–color perceived between whitish and yellowish for BM and between light and dark red for LM), aroma (smell associated with cooked meat), taste (taste associated with cooked meat), tenderness (fibers perceived during mastication), juiciness (water perceived during mastication), and overall liking (overall satisfaction and desire to eat again). The assessment was performed based on a modified five-point hedonic scale (1 = dislike extremely, 3 = neither like nor dislike, and 5 = like extremely; [24]).

### Statistical analysis

Mean values and standard deviations (SD) were calculated for each attribute. Comparisons of means for each variable between sighted and blind people were conducted using a Mann–Whitney U test [25]. Means for various types of meat (species) within each group of people were compared using Duncan’s multiple range test at 0.05 or 0.01 probability level [25]. Multivariate comparisons between scores given by the sighted and blind people were conducted using MANOVA [25]. It allows evaluating statistical significance between the sighted and blind people according to groups of traits. Multiple regression and analysis of correlations were applied for evaluation of relationships between all examined attributes and poultry meat overall appreciation. Following the regression model was used for the analyses:

yi=b0+b1×1+b2×2+…+bk×k+ei

where: y is dependent variable, x1, x2, xk are independent variables, b0 is intercept, b1, b2, bk, are partial regression coefficients, ei is error term.

The results were presented as standardized regression coefficients. In addition, evaluation of multivariate differences of the analyzed meat sensory attributes was conducted with cluster analysis. The square of Euclidian distance was adopted as a measure of distance for standardized data, and grouping of objects was conducted with the Ward’s method [25]. Results were depicted in the form of dendrograms. Analyses were performed using Statistica 12 software [26].

## Results

### Poultry meat color evaluated by the sighted panelists

**Fig 1:**
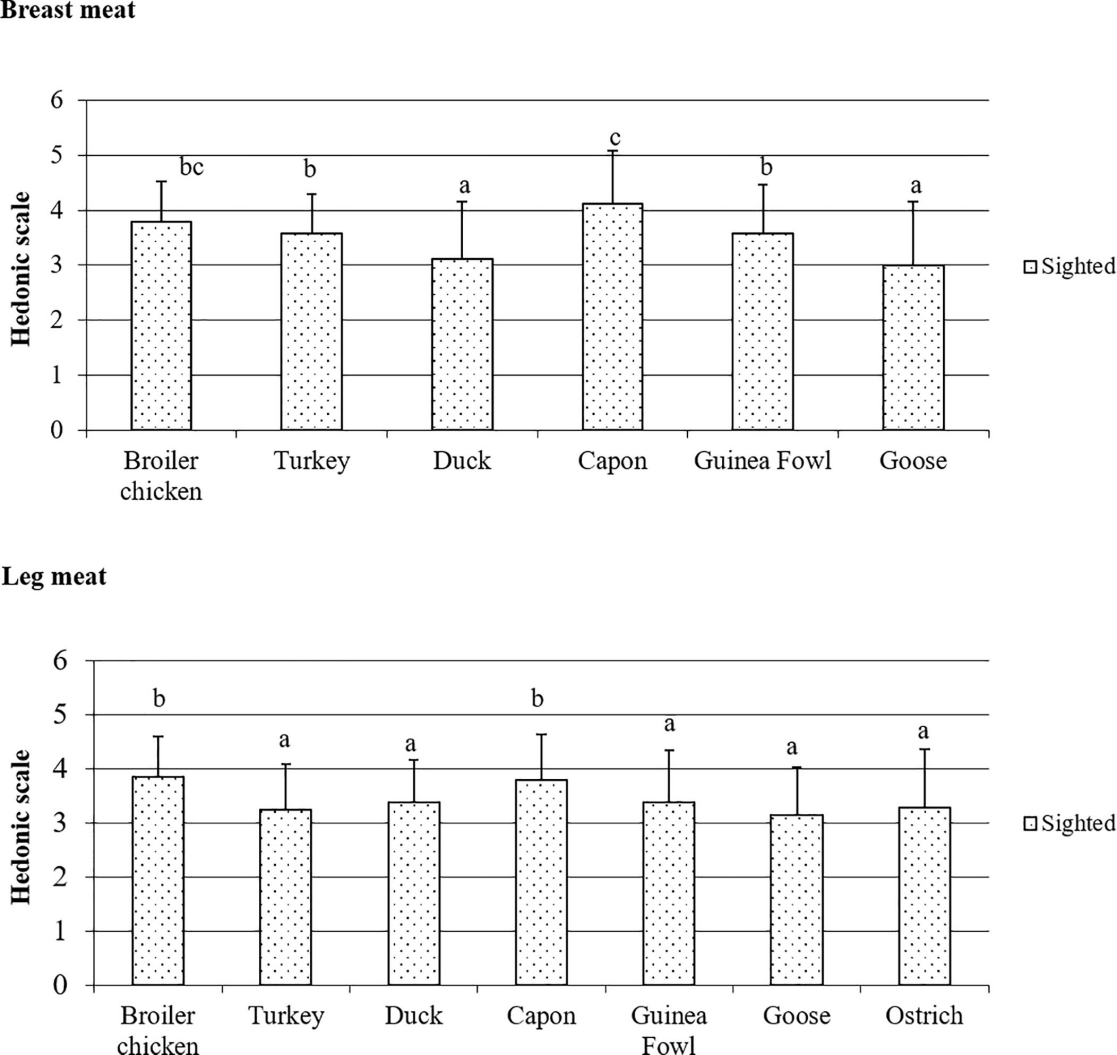
Poultry meat color based on the evaluation by the sighted panelists.

Results obtained in the study indicate different consumer preferences regarding the color of poultry meat species and muscle types (Fig 1, S1 Table). For both types of muscles, the highest scores were given by panelists to those of capon and broiler chickens; while negligibly better scores were noted for capon BM color (*P* = 0.078) and for broiler LM color (*P* = 0.062). For BM, the lowest scores (*P <* 0.05) were given to red meat from water fowl (duck and goose). Red LM received lower scores in color assessments compared to white BM, but still when comparing only red LM all species were scored similarly (*P >* 0.05), except for capon and broiler chicken.

### Poultry meat smell evaluated by the sighted and blind panelists

**Fig 2:**
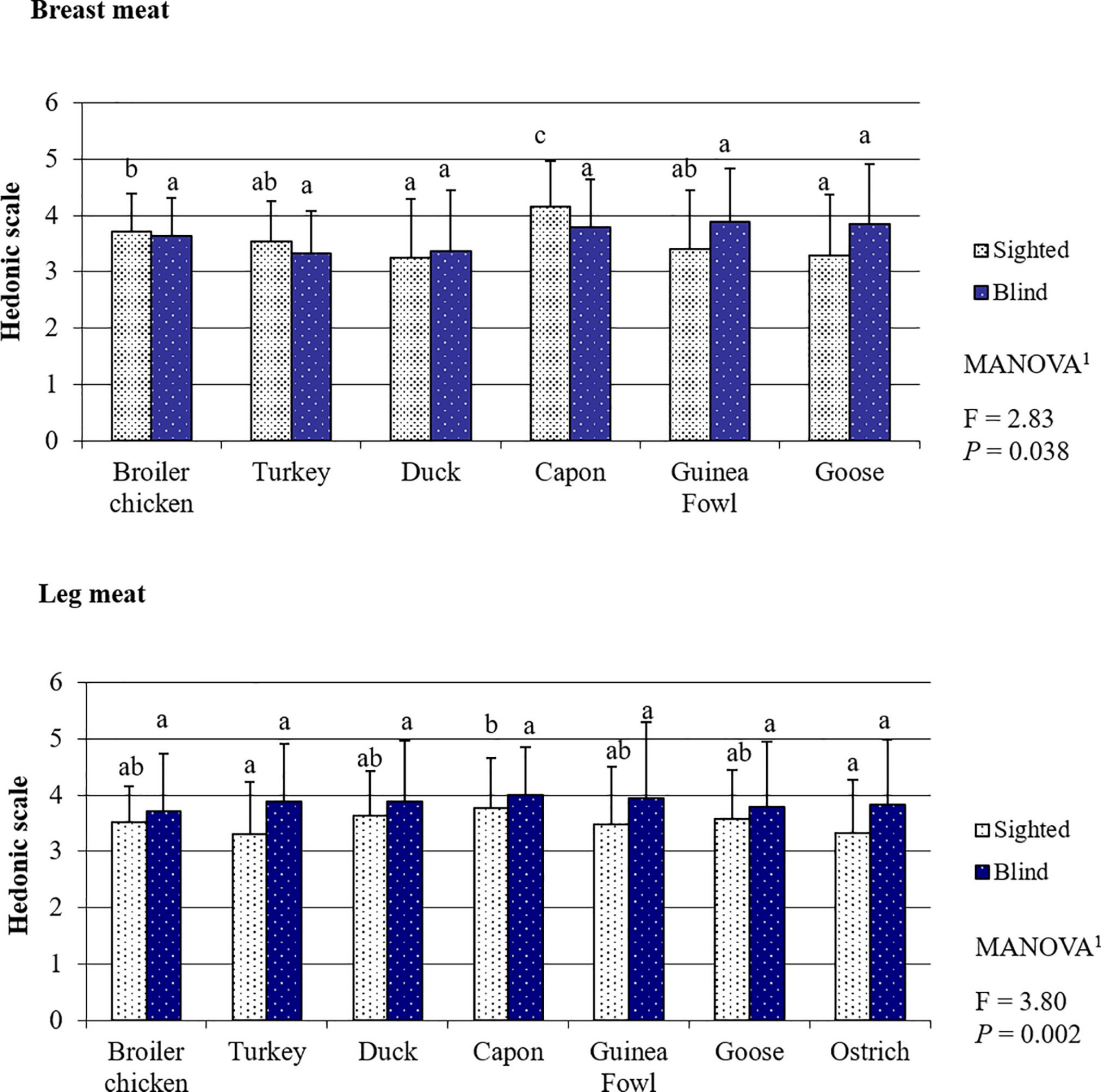
Poultry meat smell based on the evaluation by the sighted and blind panelists.

The sighted consumers assessed capon BM as having the best aroma (all BM *P >* 0.05) (Fig 2, S2 Table). For LM, the observed difference was significant only when compared to the lowest scored LM from turkey and ostrich (both *P >* 0.05). In blind panelist's opinion, the aroma of both BM and LM from all analyzed poultry species were similar (all meats *P <* 0.05). The only difference between the sighted and the blind panelists was in the perception of goose BM aroma, which was scored significantly higher by the blind persons compared to the sighted ones (*P* = 0.048). Considering scores given to aroma of all meat species collected together, the multi-way analysis of variance demonstrated significant differences in the assessment of the sighted and the blind panelists for both BM (*P* = 0.038) as well as LM (*P* = 0.002).

### Poultry meat taste evaluated by the sighted and blind panelists

**Fig 3:**
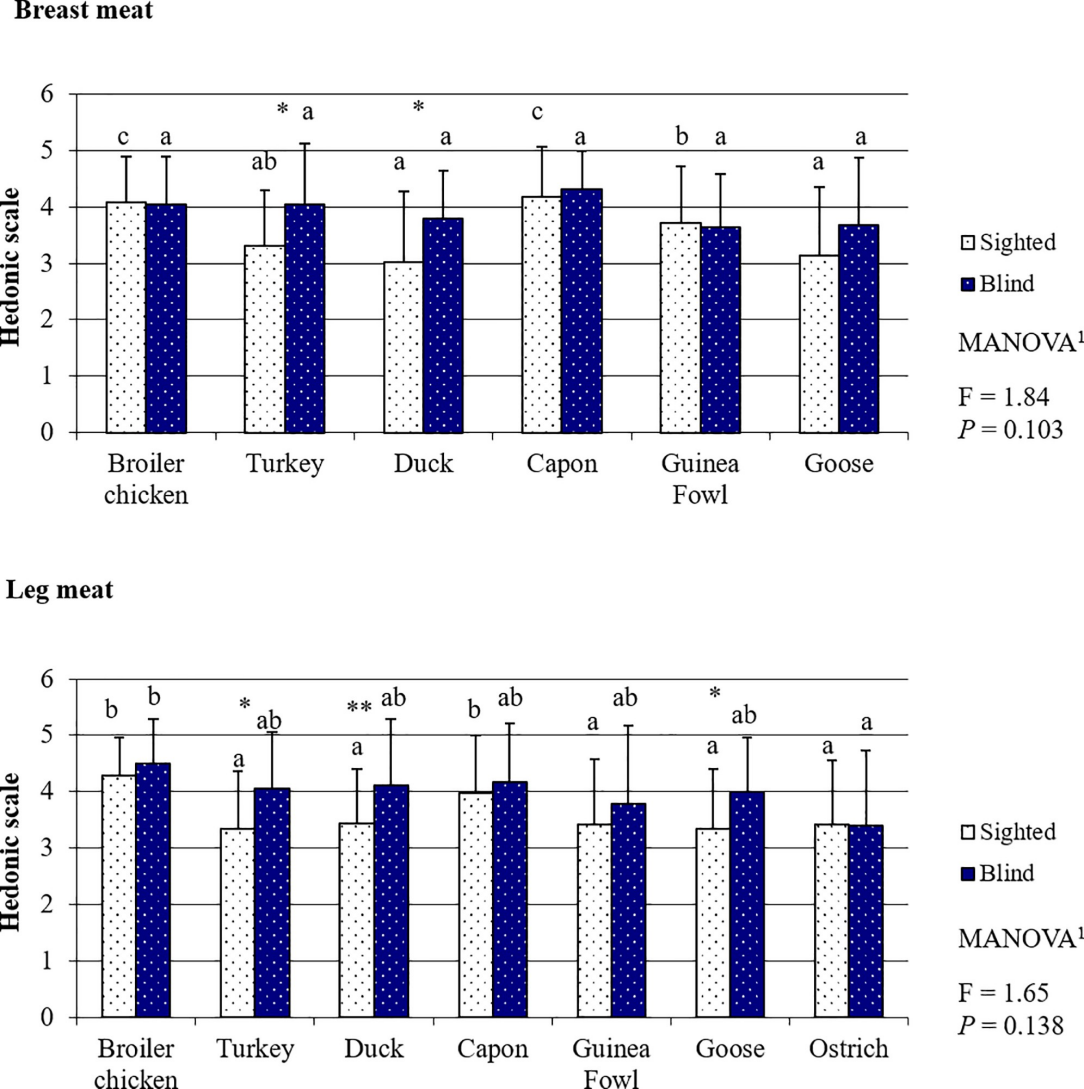
Poultry meat taste based on the evaluation by the sighted and blind panelists.

As for color and aroma, the most attractive meats to the sighted panelist in terms of taste (Fig 3, S3 Table) were BM and LM from capon and broiler chicken (all meats *P <* 0.05), whose scores did not differ significantly (BM *P* = 0.075; LM *P* = 0.059). The taste of LM from other bird species was assessed by the panelists as being similar (*P >* 0.05), whereas in terms of BM, goose and duck meat was scored significantly lower compared to the meat from Guinea fowl (duck *P* = 0.022; goose *P* = 0.037). In contrast to the sighted panelists, the blind consumers assessed the taste of BM from all poultry species at a similar level. In the opinion of the blind panelists, the least tasty LM were these from ostrich (all LMs *P >* 0.05) and Guinea fowl; however, Guinea fowl LM were assessed significantly worse only compared to the highest scored broiler chicken meat (*P* = 0.037). The blind panelists gave significantly higher scores to the taste of BM from turkey (*P* = 0.011) and goose (*P* = 0.028) and to the taste of LM from turkey (*P* = 0.020), duck (*P* = 0.010), and goose (*P* = 0.031), compared to the scores of the sighted consumers. Broiler chicken and capon meat, in taste assessment, was evaluated similarly by both groups of panelists (*P >* 0.05). Despite the many individual differences in taste assessment made for individual meat species within one group of panelists (Mann-Whitney U test) as well as the differences between the sighted and the blind panelists, the multiway analysis of variance demonstrated that the sighted and blind persons evaluated the taste of both BM (*P* = 0.103) and LM (*P* = 0.138) at a similar level.

### Poultry meat tenderness evaluated by the sighted and blind panelists

**Fig 4:**
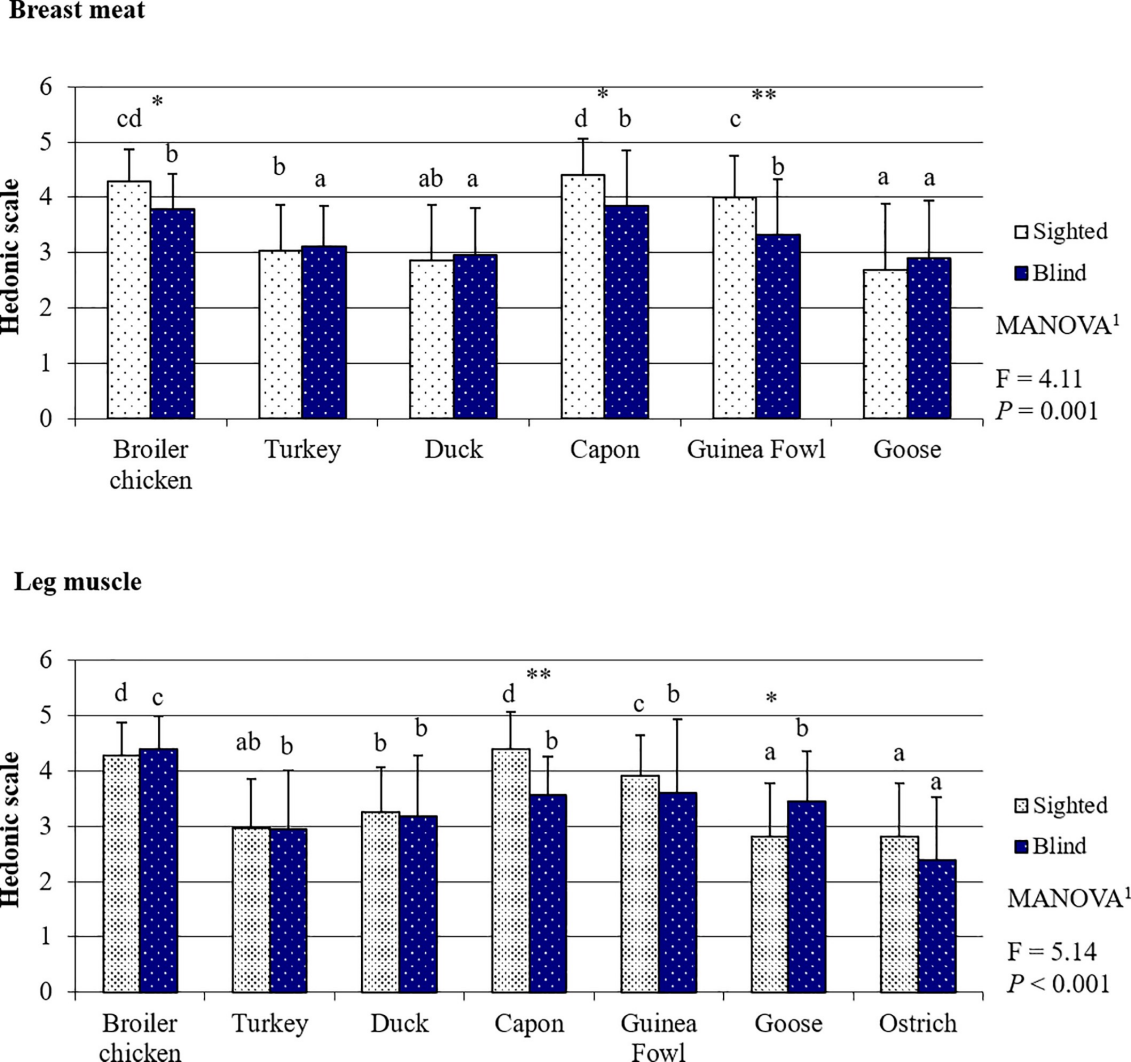
Poultry meat tenderness based on the evaluation by the sighted and blind panelists.

Both the sighted and blind panelists scored BM tenderness for the evaluated poultry species in the same order (Fig 4, S4 Table), i.e. BM from capon and broiler chicken as the most tender (both *P <* 0.05), followed by BM from Guinea fowl. The fourth in terms of tenderness turned out to be turkey BM and the fifth–duck. The worst tenderness was found for goose BM; however, in the opinion of the sighted panelists it was comparable to duck BM tenderness (*P* = 0.069), and for the blind respondents–comparable with the tenderness of BM from both duck (*P* = 0.088) and turkey (*P* = 0.055). For LM, the highest scores were again given by the sighted consumers to the muscles of capon and broiler chicken (both *P <* 0.05). In the assessment of LM tenderness, the blind panelists distinguished meat from broiler chickens as the best (all meats *P >* 0.05) and ostrich meat as the worst (all meats *P >* 0.05). Ostrich LM was also the least tender in the assessment made by the sighted panelists, but it was rated equally with LM from goose and duck. In contrast to the taste assessment in which the blind panelists in most cases gave higher scores, meat tenderness usually received significantly lower scores when evaluated by this group of consumers compared to the sighted panelists (Fig 4, S4 Table). The exception was ostrich LM, the tenderness of which was scored higher by the blind than by the sighted panelists (*P* = 0.044). As a consequence, as for meat aroma, the multiway analysis of variance demonstrated that persons with different visual capabilities compared to the blind persons evaluated the tenderness of both BM (*P =* 0.001) and LM (*P* < 0.001) differently considering all poultry species taken together.

### Poultry meat juiciness evaluated by the sighted and blind panelists

**Fig 5:**
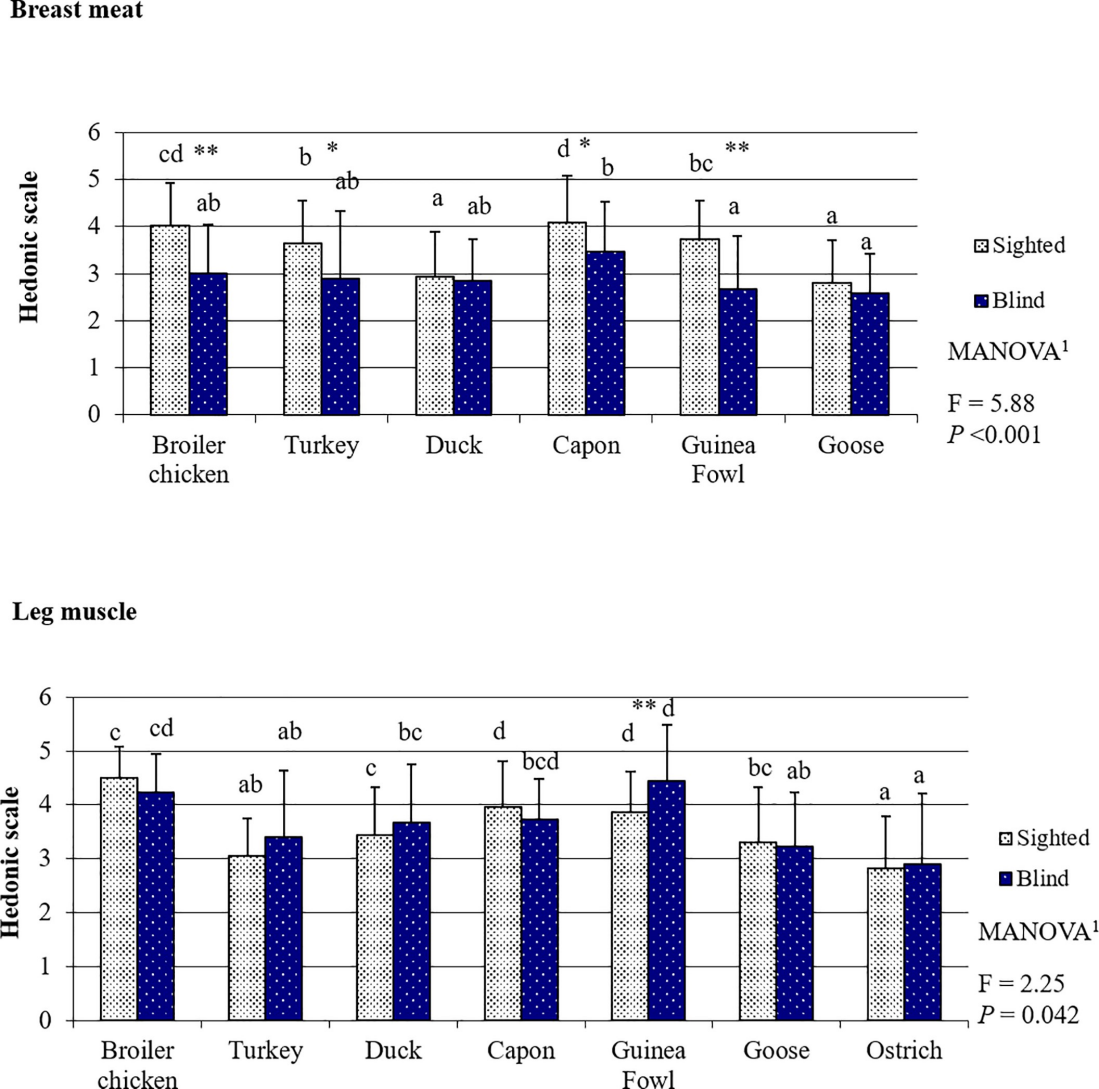
Poultry meat juiciness based on the evaluation by the sighted and blind panelists.

In the opinion of both groups of panelists, the juiciest BM were those from capon, followed by those from broiler chicken (Fig 5). The third in terms of juiciness as perceived by the sighted consumers were BM from Guinea fowl and then (with slightly worse scores) BM from turkey. In turn, in the assessment of the blind panelists, turkey BM was placed third and almost the same scores were given to duck BM. The lowest scores in BM juiciness evaluation were given by both the sighted and blind panelists to goose, but the difference between juiciness of goose BM and duck BM was insignificant when the muscles were assessed by the sighted consumers (*P* = 0.064), whereas a significant difference was demonstrated between juiciness of goose BM and capon BM in the assessment of the blind panelists (*P* = 0.021). For LM juiciness, the highest scores were given by the sighted panelists to meat from broiler chicken (*P <* 0.05), followed by similarly scored meat from capon and Guinea fowl (both *P* = 0.091). The third in terms of juiciness as evaluated by the sighted panelists were similarly scored LM from water fowl (*P* = 0.033); however, the lower scored juiciness of goose LM was in the opinion of this group of panelists comparable with juiciness of turkey LM (*P* = 0.068). The driest meat according to the sighted consumers was LM from ostrich, whose juiciness was evaluated as being similar to that of turkey LM (*P* = 0.056). The blind consumers perceived ostrich LM as the driest. The juiciness of BM was most often evaluated by the blind consumers significantly lower (Fig 5, S5 Table) than by the sighted panelists, likewise in the case of LM tenderness (Fig 4, S4 Table). Consequently, the multi-way analysis of variance demonstrated that the sighted and blind panelists evaluated BM juiciness differently (*P* < 0.001). Similar and significant results, although less intensive, were demonstrated for LM (*P* = 0.042). However, the difference in LM juiciness assessment occurred only for Guinea fowl LM which were scored significantly higher by the blind panelists (*P* = 0.003).

### Poultry meat overall liking evaluated by the sighted and blind panelists

**Fig 6:**
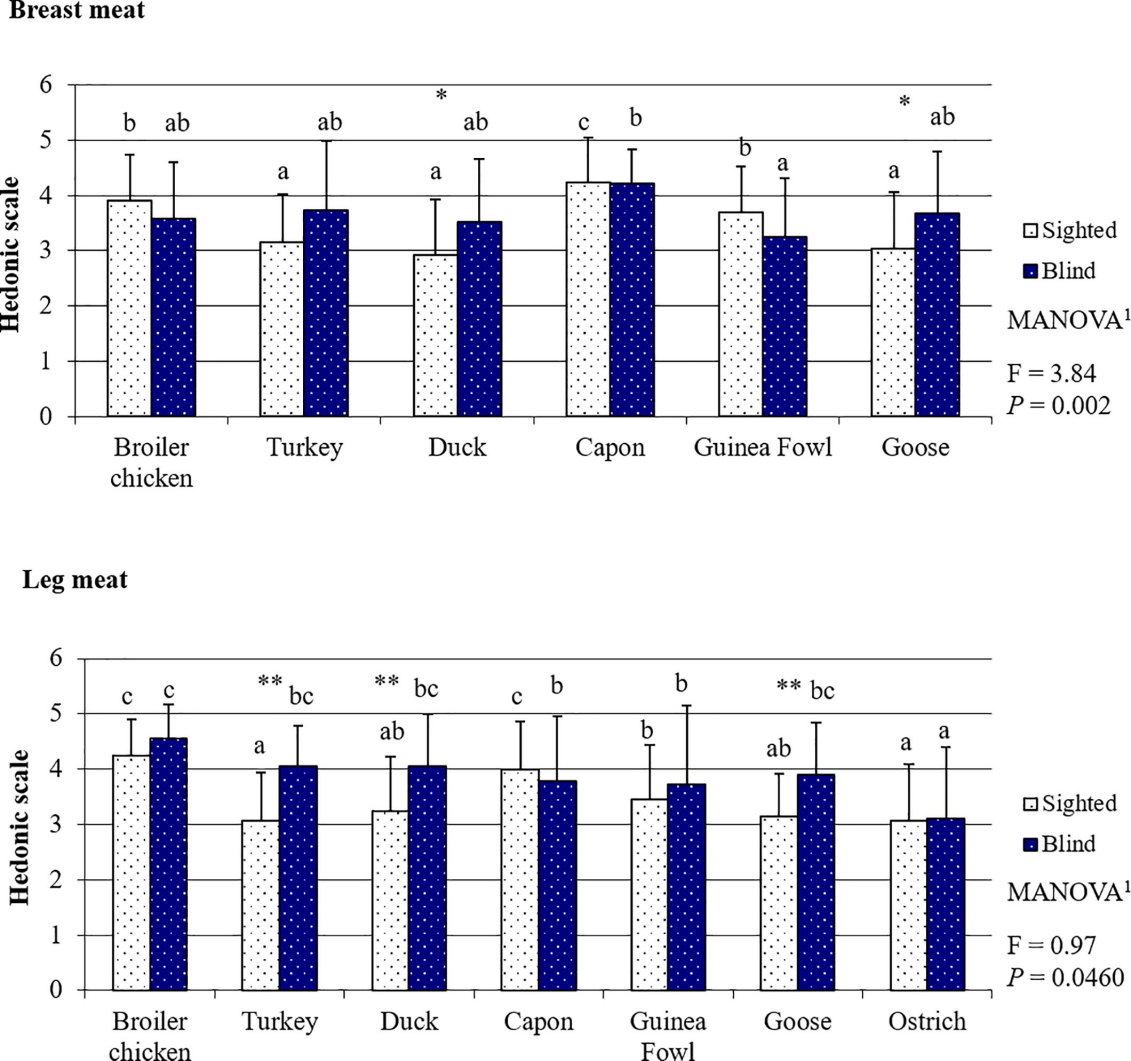
Poultry meat overall liking based on the evaluation by the sighted and blind panelists.

In BM assessment by the sighted panelists, the best in terms of overall liking (Fig 6, S6 Table) turned out to be meat from capon (all BMs *P <* 0.05), followed by broiler chicken and Guinea fowl (both *P >* 0.05). The worst scores were given by these panelists to BM from turkey, goose, and duck (all three *P >* 0.05). The blind consumers also assessed capon BM as the best in terms of overall liking. For LM, the highest scores for overall liking were given by the sighted panelists to muscles from broiler chicken and capon (all meats *P <* 0.05), whereas by the blind panelists to muscles from broiler chicken, turkey, duck, and goose. The mean score given by the blind consumers to broiler chicken LM (4.56) was also the highest score in the overall liking assessment in the whole study. Ostrich LM turned out to be the least acceptable in terms of sensory quality by both the sighted and blind panelists. The sighted consumers gave equally low scores to turkey LM and insignificantly higher scores to goose and duck LM (*P* = 0.088; *P* = 0.064, respectively). Compared to the sighted panelists, the blind consumers gave significantly higher scores in the overall liking assessment to both types of muscles from duck.

The multiple regression and correlation analyses (Table 1), describing correlations between the overall liking of meat assortment and its other analyzed attributes, demonstrated taste to have the greatest influence on the overall liking assessment made by the sighted panelists (all analyses *P* < 0.001). The overall liking of meat was also significantly affected by its tenderness and juiciness, and the least significant role was ascribed to aroma and color. For the blind panelists, the highest effect upon overall liking of meat was attributed to meat taste (all analysis *P* < 0.001). The other sensory attributes had a lesser influence on the overall liking assessment of meat; however, the sensation of juiciness had a greater impact compared to tenderness and aroma.

**Table 1:** Results of multiple regression and analysis of correlations for relationships with poultry meat overall liking (for all types of meat).

		Brest meat				Leg meat			
		Results of multiple regression		Results of analysis of correlation		Results of multiple regression		Results of analysis of correlation	
		*b*^1^	*P*^2^	*r*^3^	*P*^4^	*b*^1^	*P*^2^	*r*^3^	*P*^4^
Sighted panelists									
		R^2^ = 0.742 (with color) R^2^ = 0.734 (without color)				R^2^ = 0.742 (with color) R^2^ = 0.735 (without color)			
Color		0.097	0.006	0.586	<0.001	0.113	0.001	0.598	<0.001
Smell	*	0.111	<0.001	0.466	<0.001	0.095	0.001	0.397	<0.001
	**	0.125	<0.001			0.112	<0.001		
Taste	*	0.517	<0.001	0.812	<0.001	0.494	<0.001	0.799	<0.001
	**	0.560	<0.001			0.535	<0.001		
Tenderness	*	0.158	<0.001	0.649	<0.001	0.163	<0.001	0.653	<0.001
	**	0.164	<0.001			0.193	<0.001		
Juiciness	*	0.174	<0.001	0.635	<0.001	0.205	<0.001	0.663	<0.001
	**	0.179	<0.001			0.207	<0.001		
Blind panelists									
		R^2^ = 0.578				R^2^ = 0.725			
Smell		-0.012	0.852	0.222	0.018	-0.022	0.717	0.510	<0.001
Taste		0.730	<0.001	0.755	<0.001	0.812	<0.001	0.844	<0.001
Tenderness		0.001	0.988	0.245	0.009	0.025	0.643	0.323	<0.001
Juiciness		0.095	0.158	0.315	0.001	0.108	0.045	0.391	<0.001

^1^Standardized partial coefficient of multiple regression

^2^*P*-value based on multiple regression analysis

^3^Coefficient of correlation

^4^*P*-value based on analysis of correlation

* Regression of all analyzed traits for sighted panelists

** Regression of seeing panelists analysis without color

### Generalized distance by multi-variate analyses for all meat species and between the sighted and blind panelists based on all groups of traits

**Fig 7:**
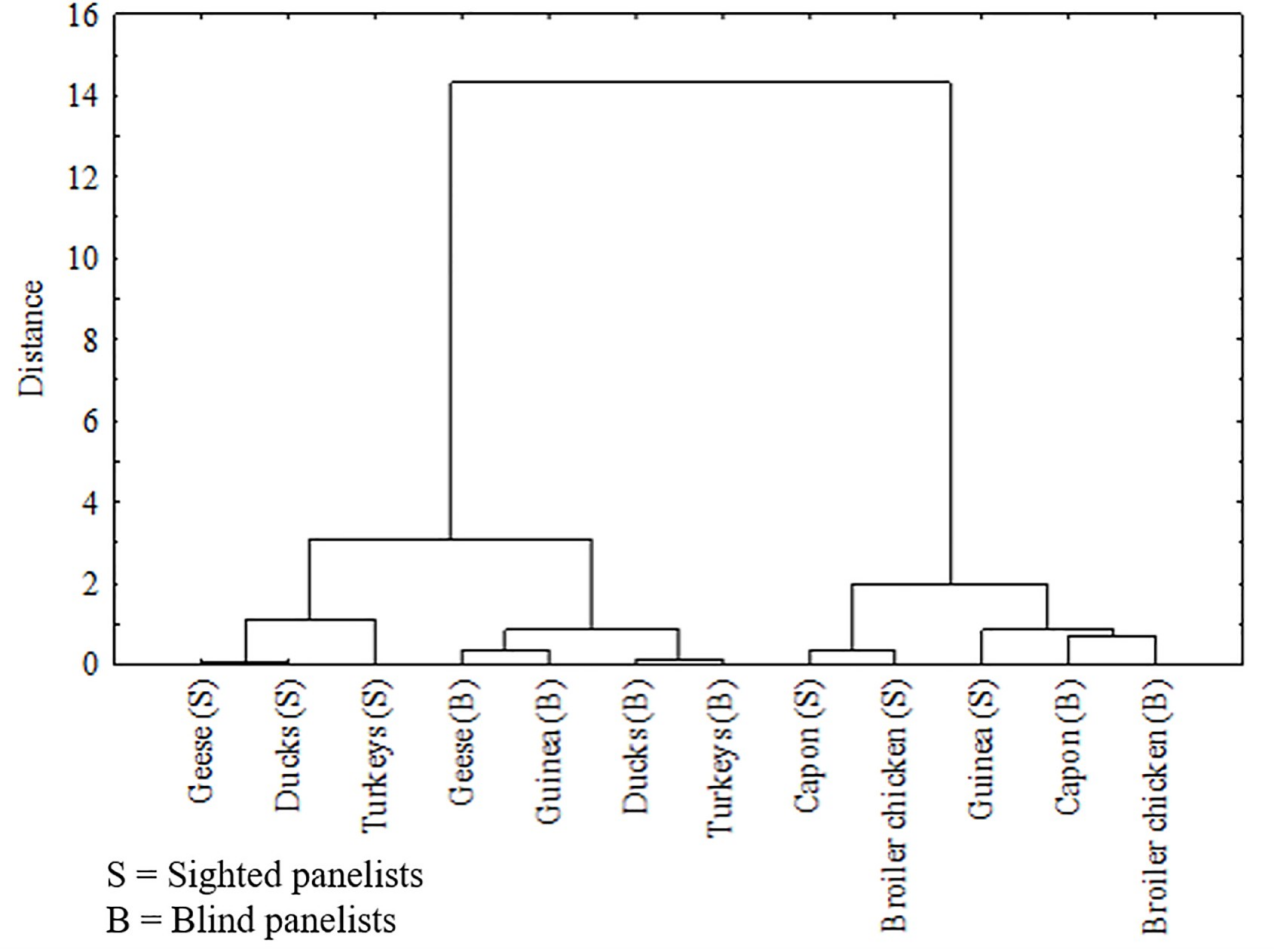
Dendrograms based on cluster analysis using Ward’s method using five examined traits poultry breast meat, ie. smell, taste, tenderness, juiciness and overal liking.

**Fig 8:**
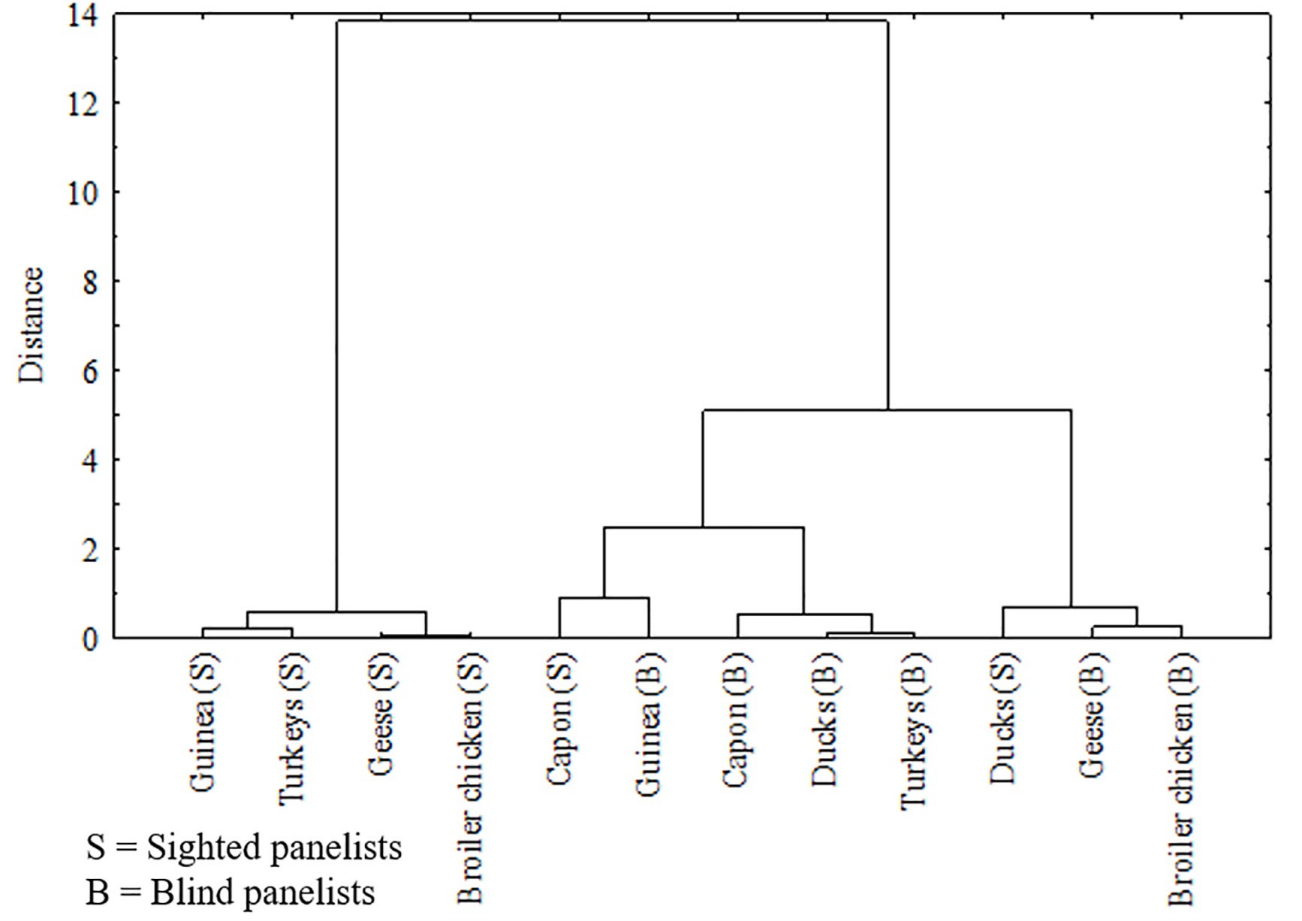
Dendrograms based on cluster analysis using Ward’s method using five examined traits poultry leg meat, ie. smell, taste, tenderness, juiciness and overal liking.

Results of the MANOVA and pairwise comparisons of all sensory attributes of the analyzed meat species are presented in Fig 7 and Table 2 for BM and in Fig 8 and Table 3 for LM. Based on MANOVA, in the opinion of the sighted panelists, the most similar BMs in terms of sensory impressions were muscles from duck and goose (*P* = 0.690) as well as muscles from capon and broiler chicken (*P* = 0.051). A significantly lesser similarity was found between BM from Guinea fowl and broiler chicken (*P* = 0.008) and between turkey and duck BM (*P* = 0.008). In the opinion of the blind panelists, the most similar BMs in terms of sensory attributes were those of turkey and duck (*P* = 0.920), followed by those of duck and goose (*P* = 0.521). The BMs of capon and broiler chicken, which received the highest scores in sensory quality assessment, were also classified to one homogenous group but with a lesser similarity (*P* = 0.326). Noteworthy is the fact that based on scores given by the blind panelists, compared to those given by the sighted consumers, we were unable to indicate poultry species differing significantly (*P* > 0.001) between each other in terms of all sensory attributes of BM assessed together.

**Table 2:** Results of MANOVA (Wilks’s test) and multi-variate (based on all traits) pairwise comparisons based on F-test for traits of breast meat.

**Sighted panelists**						
MANOVA: F = 10.14, *P* < 0.001						
Multi-variate pairwise comparisons *P*-values (below of diagonal)\F statistics (above of diagonal)						
	Broiler chicken	Turkey	Duck	Capon	Guinea fowl	Goose
Broiler chicken		17.75	18.98	3.31	2.29	18.33
Turkey	<0.001		3.84	25.32	11.75	6.02
Duck	<0.001	0.003		24.99	11.03	0.61
Capon	0.051	<0.001	<0.001		5.07	20.19
Guinea fowl	0.008	<0.001	<0.001	<0.001		11.71
Goose	<0.001	<0.001	0.690	<0.001	<0.001	
**Blind panelists**						
MANOVA: F = 1.71, *P* = 0.019						
Multi-variate pairwise comparisons *P*-values (below of diagonal)\F statistics (above of diagonal)						
	Broiler chicken	Turkey	Duck	Capon	Guinea fowl	Goose
Broiler chicken		2.75	2.84	1.21	1.62	3.60
Turkey	0.036		0.28	2.64	1.81	1.52
Duck	0.031	0.920		3.00	1.55	0.86
Capon	0.326	0.041	0.025		2.65	2.37
Guinea fowl	0.182	0.139	0.204	0.041		1.12
Goose	0.011	0.212	0.521	0.061	0.372	

**Table 3:** Results of MANOVA (Wilks’s test) and multi-variate (based on all traits) pairwise comparisons based on F-test for traits of leg meat.

**Sighted panelists**							
MANOVA: F = 9.23, *P* < 0.001							
Multi-variate pairwise comparisons *P*-values (below of diagonal)\F statistics (above of diagonal)							
	Broiler chicken	Turkey	Duck	Capon	Guinea fowl	Goose	Ostrich
Broiler chicken		31.40	15.39	5.91	6.87	22.50	26.45
Turkey	<0.001		1.86	19.54	11.29	1.54	0.75
Duck	<0.001	0.109		12.96	5.47	1.52	4.57
Capon	<0.001	<0.001	<0.001		3.35	19.55	23.76
Guinea fowl	<0.001	<0.001	<0.001	0.008		9.69	15.37
Goose	<0.001	0.183	0.191	<0.001	<0.001		2.62
Ostrich	<0.001	0.586	0.001	<0.001	<0.001	0.029	
**Blind panelists**							
MANOVA: F = 2.94, *P* < 0.001							
Multi-variate pairwise comparisons *P*-values (below of diagonal)\F statistics (above of diagonal)							
	Broiler chicken	Turkey	Duck	Capon	Guinea fowl	Goose	Ostrich
Broiler chicken		9.20	5.11	8.72	4.83	3.79	10.73
Turkey	<0.001		0.18	1.29	2.12	0.63	1.90
Duck	0.002	0.968		0.93	1.78	0.54	2.13
Capon	<0.001	0.294	0.476		2.34	1.52	3.28
Guinea fowl	0.002	0.091	0.148	0.066		3.56	4.56
Goose	0.009	0.677	0.742	0.213	0.012		2.13
Ostrich	<0.001	0.124	0.089	0.018	0.003	0.089	

Considering the scores given in the sensory assessment by the sighted panelists, broiler chicken LM differed significantly from all other poultry species (*P* < 0.001). Ostrich and turkey LM (*P* = 0.586) as well as duck and goose LM (*P* = 0.191) were classified to the same homogenous group. A lesser similarity was found between turkey and goose LM (*P* = 0.183) and duck LM (*P* = 0.109) and between ostrich and goose LM (*P* = 0.191) and duck LM (*P* = 0.001). In the assessment made by the blind panelists, broiler chicken LM differed significantly (*P* < 0.001) from LM from turkey, capon, and ostrich in terms of the analyzed sensory attributes, likewise in the assessment of the sighted consumers. The most similar to each other turned out to be LM from turkey and duck (*P* = 0.968) with goose LM (*P* = 0.677), as well as goose and duck LM (*P* = 0.742), and capon and duck LM (*P* = 0.476).

On the basis of dendrogram (Fig 7) and matrix of multivariate distances (results not presented) we can conclude that differences between normal sighted and blind people according to variables of sensory evaluation of BM were the biggest for guinea fowl (*P* < 0.001). Slightly smaller differences were observed for other birds, the smallest differences were observed for capon (*P* = 0.011). The biggest multivariate differences between normal sighted and blind people according to variables of sensory evaluation of LM (Fig 8) were detected for broiler chicken (*P* < 0.001) and geese (*P* < 0.001). Moreover, big differences were observed for guinea fowl (*P* = 0.006), while the smallest differences for capon. The multivariate differences between normal sighted and blind people according variables of sensory evaluation were bigger for LM in comparison with BM.

## Discussion

### Sensory quality of poultry meat assessed based on sensory perceptions of the sighted and blind panelists

The results clearly show that the sensory perception of poultry meat by the sighted and blind people was completely different. This is mainly indicated by the fact that, apart from taste (Fig 3), the assessment of the other sensory attributes differed between the groups of panelists (Figs 2 and 4–6). The lack of differences in the perception of the taste of meat of most poultry species suggests that visual disability has no or little effect on this sense. Sipos et al. (2011) [27] have conducted a similar study concerning the assessment of the sensory quality of five apple varieties. They demonstrated that blindness affected the overall sensory acceptability of the fruits; however, differences between assessments made by the sighted and blind panelists concerned mainly such attributes as: flavor, texture, and perception of size, and to a lesser extent the taste of fruits. In our study, it was also the perception and preference for such sensory attributes as tenderness and juiciness–both perceived with the sense of touch–that caused differences in the sensory assessment of meat between the sighted and blind persons. These results are in line with the preceding observations of Gagnon et al. (2013) [28]. These authors indicated that blind persons have lower taste sensitivity in comparison to sighted persons. This is primarily associated with training. The taste system is susceptible to plasticity induced by training, similarly to other senses. Bilyk et al. (2009) [29] demonstrated that in their everyday life, blind people encounter strong obstacles in such activities as buying food, preparing meals or eating in restaurants. Thus, the diet of blind persons is typically less varied, and therefore the training of flavor sensations is weaker than in the case of sighted people. On the contrary, even the need of using Braille script results in the touch being the sense heavily trained by the blind persons and it can be better used by them to identify items than sighted persons. According to Wong et al. (2011) [30], visual impairment is compensated for by, primarily, increased sensitivity in the sense of touch. This stems from the fact that when the brain does not receive visual stimuli, structural and functional reorganization proceeds in those of its regions which use other sensory modalities and areas intermediating in the integration of sensory information needed for the perception of the world, including especially identification of foods [31,32,33]. It is also a natural mechanism which would be developed by, e.g., blind or partially sighted animal species. For instance, to compensate for their visual disability star-nosed moles (*Condylura cristata*) have throughout evolution developed twenty-two fleshy snout appendages which are used as a touch organ, known as Eimer's organs [34]. This provides indisputable proof that visual disability is to a large extent compensated for by the sense of touch.

The assessment of food through the sensation of touch will, however, occur only when two other senses fail or when their perception is insufficient to make a final decision. People initially evaluate food through the sense of sight. If, however, the visual sensations are excluded, first the sense of smell and then the sense of touch are engaged. The last stage of assessment includes eating; hence, the possibility of evaluating taste “*If taste is the gatekeeper*, *the sense of smell is the sentinel*, *evaluating the food for danger before it enters the mouth*” [35]. Above all, smell enables the rejection of spoiled food. The flavor of meat derives mainly from saturated or unsaturated fatty acids. Heat treatment, of meat causes unsaturated fatty acids contained in meat to induce oxidation of the generated hydrogen peroxides, which are ultimately degraded via free radical mechanisms eventually resulting in aldehydes, unsaturated alcohols, ketones and lactones, which are responsible for the aroma [36]. Therefore, meat aroma is determined by fat content, fatty acid composition, and the region of tissue this fat occurs in. This explains the significantly greater differences between the sighted and blind persons in the perception of the aroma of cooked LM (*P = 0*.*002*) and BM (*P = 0*.*038*). Compared to BM, LM contains twice as much fat and ca. 10% more unsaturated fatty acids, due to which its aroma is more intensive and easier to assess [37]. Furthermore, Koutsoklenis and Papadopoulos (2011) [38] suggested that blind people use smells as identification and location-aiding signals far more frequently. As a consequence, the use of smell, like in the case of touch, is much more intensively trained by blind persons than by sighted people. This explains the differences in the perception of meat smell between sighted and blind people. Perhaps, the blind persons detect aromatic fat derivatives contained in meat with higher intensity, as they can remain undetected by sighted people when present in low concentration.

Earlier findings reported by Sipos et al. (2011) [27] confirmed that the sensory testing of food aimed at determining the effect of visual impressions on the other senses is reliable mainly when conducted by blind persons, because the involvement of blindfolded sighted persons in consumer or semi-consumer tests ended in highly diverse results in terms of the sensory assessment: “*blindfolding caused uncertainty and disturbed perception*, *leading to inconsistent judgements*”. In our study, the variability of scores given to sensory attributes by the blind and sighted panelists was in most cases at a similar level. Interesting were results of the analysis of evaluation sheets of two blind panelists–monozygotic twins (brothers), whose scores were almost identical, although they could not communicate with each other during testing. Of course, a single case of twins does not produce any explicit finding, but it is worth mentioning as it has been demonstrated several times that the perception of sensory impressions of food products, including mainly their taste and aroma, is also partly affected by the genetic differences in the chemosensory pathways [39,40]. Considering this fact, we chose persons of the same nationality for our study, which could be the reason behind the relatively low variability of the results achieved.

### Ranking of poultry meat sensory quality

The range of poultry meat used in our study deliberately included meat which is known to most of the panelists from their everyday diets, as well as meat which they consume occasionally or which they had never tried or which they had tried sporadically. The frequency of consumption of a given meat species depends on e.g. the origin of the panelists, and in this study this was adjusted to current trends observed in Poland. The everyday diet of Poles includes mainly chicken and turkey meat, whereas meat from water fowl is consumed occasionally (usually in the holiday season), while meat from capon, ostrich, and Guinea fowl is an exclusive, expensive, and difficult to buy product. The habitual flavor profile was, therefore, the likely cause of the highly evaluated sensory quality of broiler chicken meat by both the sighted and blind consumers. In addition, out of all the species analyzed, broiler chickens are slaughtered the earliest, their meat is tender and delicate. In turn, although perceived as a relatively rich source of protein and recommended as a dietetic product, turkey meat is poor in fat as a carrier of taste. In addition, the long fattening period of birds (15 wk for hens and 20–22 wk for toms) makes the meat significantly harder in instrumental evaluation compared to the meat of broiler chickens [41,42]. Results obtained in our study confirm the low sensory value of turkey meat and therefore point to the necessity of counteracting these practices.

An interesting finding from this study is the very high assessment of the sensory quality of both BM and LM from capon. Especially the sighted panelists praised capon meat for most of the analyzed sensory attributes and indicated BM to be the best in terms of the overall liking compared to all other analyzed meats (Fig 6). In many cases, scores given to capon meat by the panelists exceeded scores given to broiler chicken meat. This finding is by far the most interesting as it confirms the advisability of caponization as a procedure aimed at improving the quality of meat of roosters of local breeds. Amorim et al. (2016) [43] have recently demonstrated that consumers prefer meat from capons and evaluate their sensory quality much more highly compared to meat of roosters of the same breeds, broiler chickens, and also free range chickens. This is because caponization contributes to the accumulation of higher amounts of intramuscular fat, improves the lipid profile of meat, and reduces the growth of muscle fibers, owing to which meat becomes more tender, juicy, and tasty. Today, capon meat is the most expensive poultry meat and one of the most expensive meats in general. This results from the necessity of conducting the caponization procedure, the long fattening period, specific feeding, and the necessity of choosing a specified of roosters. Study results may suggest that the explicit preferences of consumers should stimulate future research on the improvement of capon production technology that would enable the reduction of production costs and make this product more accessible to a wider group of consumers, including lower-income social groups.

As for capon, a relatively expensive and unpopular poultry meat is Guinea fowl. Apart from the African countries, which are the homeland of this species, and France where the production of meat-type birds has been initiated and developed [44], in other regions of the world Guinea fowls are typically imported or kept on small household farms. Together with broiler chickens and turkeys, Guinea fowls belong to the order of the Galliformes; hence, their meat includes white BM and red LM. The few studies on Guinea fowl meat quality demonstrate that its BM is characterized by a higher protein content and a lower fat content compared to broiler chicken BM [45]. In turn, Nsoso et al. (2008) [46] demonstrated the protein content in Guinea fowl BM to be significantly higher than in LM, and the contents of fat and water to be similar in both types of muscles. However, the BM and LM from Guinea fowl differ significantly in their fatty acid profile, including especially high contents of mono- and polyunsaturated fatty acids in LM, and a higher content of n-6 fatty acids and a lower ratio of polyunsaturated to saturated fatty acids in BM. These differences were, probably, the cause of differences in scores given in the sensory assessment of the breast and leg muscles, especially by the blind panelists who rely mainly on the sense of touch. The blind consumers especially praised the juiciness of Guinea fowl LM (4.44) compared to BM (2.68), which could be due to the high content of unsaturated fatty acids, the long-chain ones in particular, in these muscles. The high content of unsaturated fatty acids is positively correlated with high sensory scores, because it creates the sensation of a more soft and delicate meat, compared to the more compact and harder tissues with a high content of saturated fatty acids [47]. Insignificantly higher scores given in the sensory quality assessment of Guinea fowl BM by the sighted panelists resulted from a relatively high evaluation of meat color. This not only confirms the role of sight in food assessment, but also indicates consumer preferences for the white and not red muscles.

The increased consumption of goose and duck meat in the holiday seasons may suggest high sensory values of these products. Unfortunately, water fowl meat was not preferred in our study, especially by the sighted panelists who classified BM and LM from both these species in terms of their sensory quality to the same group, being different from the other analyzed meat (Figs 7 and 8). The blind panelists scored goose and duck meat higher, particularly the taste of LM (Fig 3). The low scores given by the sighted panelists in the overall liking assessment could result from low scores for meat color, mainly for BM (Fig 1), which–being a red-type meat–distinguished itself from the other white muscles. Previous studies on the sensory profile of water fowl meat have usually presented different results indicating goose and duck meats as products of high sensory values [48]. The likely reason for these differences is that this type of meat is scored higher in the absence of a direct comparison with meats from other poultry species, the galliformes in particular. In addition, in our study, meat samples were cooked without skin and subcutaneous fat to make the analyzed range more unified. In most studies addressing the sensory profile of goose and duck meat, analyzes have been conducted for fillets with skin and subcutaneous fat or for products made of fillets, e.g. spickgans (smoked pectoral meat from goose with skin and subcutaneous fat) [49], whereas meat from water fowl and especially its BM devoid of skin and subcutaneous fat has a relatively low content of fat and is a thick-fiber tissue [50]. Hence, special attention should be paid to the fact that our results concern exclusively cooked lean meat from geese and ducks, whereas the sensory profile of commonly used products containing other tissues (subcutaneous fat, skin) may be evaluated significantly higher.

By far the worst scored meat in our study was ostrich meat. Its low overall liking resulted mainly from low scores given to its tenderness (Fig 4) and juiciness (Fig 5). This is probably because ostriches need a long time to reach slaughter maturity (12–15 months). In addition, their meat is built of very thick fibers owing to their high body weight and the intensive work of their LM which enables them to run very fast. According to Horbańczuk, and Wierzbicka (2017) [51], this meat is currently gaining in popularity among consumers seeking an alternative to a standard diet, who value its low fat content, high content of polyunsaturated fatty acids, beneficial ratio of n-6/n-3 fatty acids, and significantly higher content of iron (heme form) compared to poultry meat and beef. Of course, in terms of the nutritive value of meat, all the mentioned traits are highly beneficial to consumers, but do not always correspond with the high sensory values of meat. As in the case of, e.g. turkey meat, the low fat content has a rather negative effect on taste, tenderness, and juiciness. There is no research available that would compare the sensory quality of ostrich meat with that of other poultry species, and this impairs discussion of our results. However, some reports indicate that the addition of comminuted ostrich meat to meat products improves their sensory values [52]. This suggests that ostrich meat may be a desirable component of cured meat products and processed meat products, but is not preferred as a cooked meat serving.

## Conclusion

In summary, that the inclusion of the blind persons onto a panel evaluating the sensory profile of food–in our case of poultry meat–affords new methodological possibilities for the quality assessment of food products. In addition, our study results indicate many differences in the perception of sensory impressions used in food assessment between sighted and blind consumers. They also confirm the fact that blindness is compensated for by other senses responsible for food assessment. Although this study constitutes the first attempt at carrying out a sensory panel for products of animal origin with the participation of completely blind persons, the results are significant in terms of providing information on the quality of the product without its falsification with a visual impression. This is of great importance with regards to the pressure put by commercial enterprises on consumers through advertising, the positive reception of which oftentimes stems from the use of modern graphic techniques, and not the quality of the product itself. Finally, our study proves that the unlimited possibility for the visual assessment of food products affects the perception of other sensations. This offers significant opportunities not only for science and industry, but also for visually disabled persons as they may use their different perception capabilities in practice. In the future, however, a specific schedule of training for blind panelists should be elaborated in regard of particular groups of food products. The assessment of the sensory quality of cooked meat did not involve the use of the sense of hearing, which is perceived as the sharpest sense for blind persons, while sound analysis may be very useful in the assessment of such food products as bakery products, chips or some confectionery products. It would also be interesting to conduct sensory testing by blind panelists in other than hedonic scale models, e.g. in triangle tests.

Results obtained in this study clearly show that the most preferred poultry meat in terms of the sensory profile were breast muscles (BM) from broiler chicken and capon. Consumer preferences concerning sensory impressions are therefore ambiguous and are in part consistent with economic preferences. Our study results indicate that–considering consumer preferences–reorganization of the poultry meat market should be aimed at developing a less expensive technology for capon production. In addition, our results confirmed the low sensory quality of turkey meat, which however needs to be addressed in future studies and development of a production strategy to counteract this phenomenon is urgently required.

## Supporting information

S1 Fig Preparation, packaging and cooking of poultry meat: Broiler chicken = 1, turkey = 2, duck = 3, capon = 4, guinea fowl = 5, goose = 6 and ostrich = 7. (TIF)

S2 Fig Poultry meat samples cooked with the sous–vide method and prepared for consumer testing. (TIF)

S3 Fig Blind panelist reading the assessment card prepared in Braille. (TIF)

S1 Table Data for statistical means and variability for poultry meat color evaluation. (DOC)

S2 Table Data for statistical means and variability for poultry meat smell evaluation. (DOC)

S3 Table Data for statistical means and variability for poultry meat taste evaluation. (DOC)

S4 Table Data for statistical means and variability for poultry meat tenderness evaluation. (DOC)

S5 Table Data for statistical means and variability for poultry meat juiciness evaluation. (DOC)

S6 Table Data for statistical means and variability for poultry meat overall liking evaluation. (DOC)

S1 Dataset Original raw data set. (XLS)
