# Supplementary figures and images for: Sensory evaluation of poultry meat: A comparative survey of results from normal sighted and blind people
Source: PLoS One. 2019 Jan 30;14(1):e0210722. doi: 10.1371/journal.pone.0210722 (PMC6353138; doi:10.1371/journal.pone.0210722)

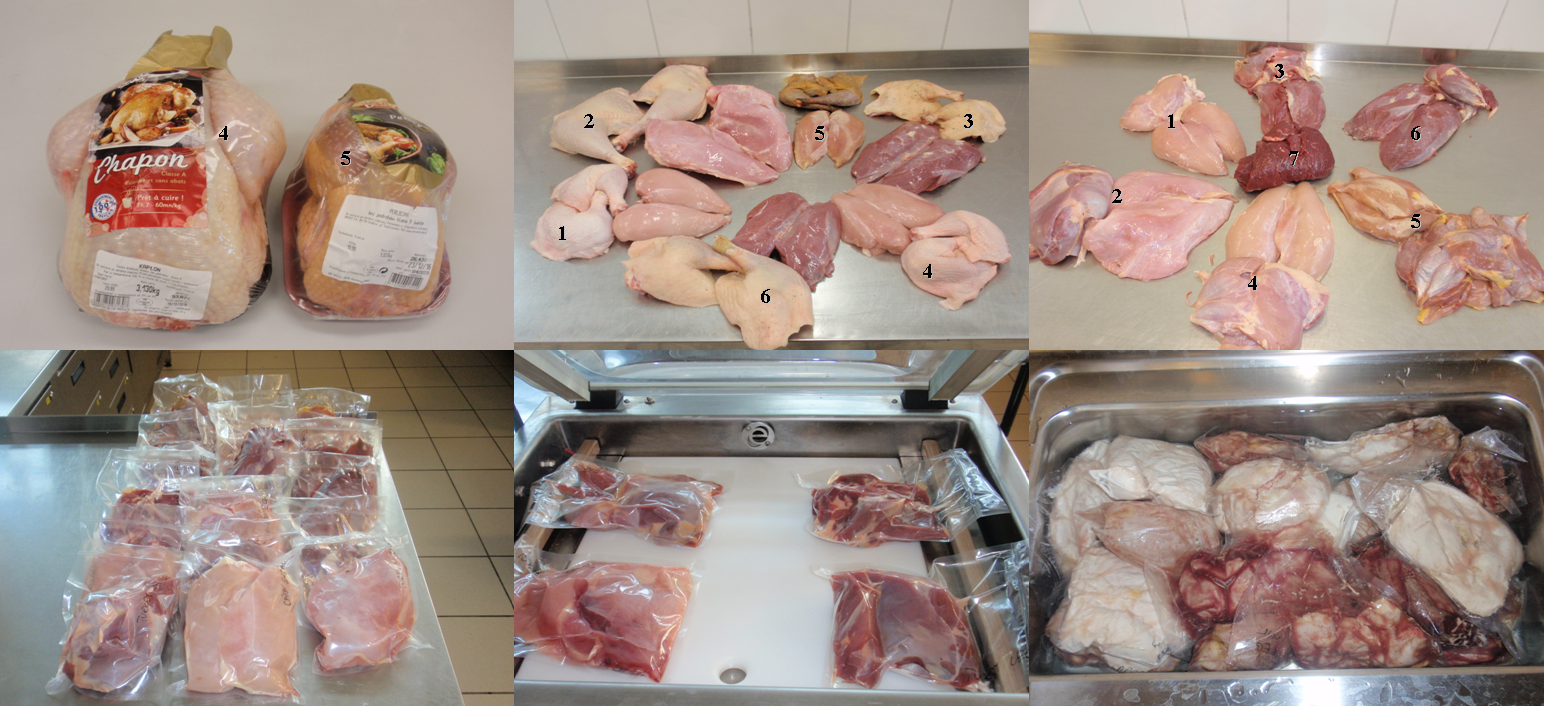

Supplement: S1 Fig — (TIF) [file pone.0210722.s001.tif]

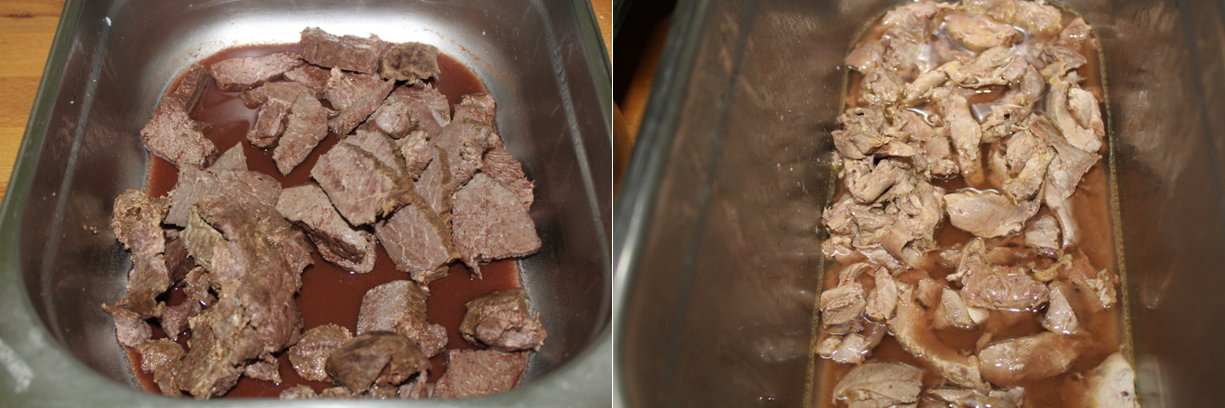

Supplement: S2 Fig — (TIF) [file pone.0210722.s002.tif]

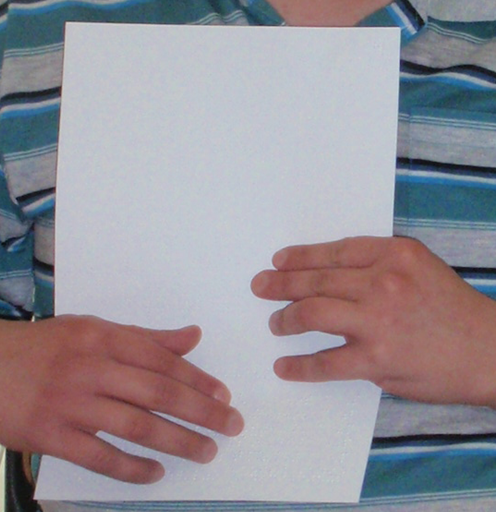

Supplement: S3 Fig — (TIF) [file pone.0210722.s003.tif]
