# Supplementary material for: Sensory evaluation of poultry meat: A comparative survey of results from normal sighted and blind people
Source: PLoS One. 2019 Jan 30;14(1):e0210722. doi: 10.1371/journal.pone.0210722 (PMC6353138; doi:10.1371/journal.pone.0210722)
Supplement: S1 Table — (DOC) [file pone.0210722.s004.doc]

**S1 Table** Data for statistical means and variability for poultry meat color evaluation

| Type of meat | Sighted panelists | |
| --- | --- | --- |
|  | Mean | SD |
| Breast meat | | |
| Broiler chicken | 3.79^bc^ | 0.74 |
| Turkey | 3.57^b^ | 0.73 |
| Duck | 3.12^a^ | 1.04 |
| Capon | 4.12^c^ | 0.97 |
| Guinea fowl | 3.57^b^ | 0.90 |
| Goose | 3.00^a^ | 1.15 |
| Leg meat | | |
| Broiler chicken | 3.84^b^ | 0.76 |
| Turkey | 3.24^a^ | 0.84 |
| Duck | 3.37^a^ | 0.80 |
| Capon | 3.78^b^ | 0.86 |
| Guinea fowl | 3.37^a^ | 0.96 |
| Goose | 3.14^a^ | 0.89 |
| Ostrich | 3.29^a^ | 1.06 |

^a-c^ Different letters within columns indicate significant differences based on Duncan’s multiple range test at 0.05 level of significance
