# Supplementary material for: Sensory evaluation of poultry meat: A comparative survey of results from normal sighted and blind people
Source: PLoS One. 2019 Jan 30;14(1):e0210722. doi: 10.1371/journal.pone.0210722 (PMC6353138; doi:10.1371/journal.pone.0210722)
Supplement: S2 Table — (DOC) [file pone.0210722.s005.doc]

**S2 Table** Data for statistical means and variability for poultry meat smell evaluation

| Type of meat | Sighted panelists | | Blind panelists | | *P*^1^ |
| --- | --- | --- | --- | --- | --- |
|  | Mean | SD | Mean | SD |  |
| Breast meat | | | | |  |
| Broiler chicken | 3.71^b^ | 0.68 | 3.63^a^ | 0.68 | 0.628 |
| Turkey | 3.53^ab^ | 0.73 | 3.32^a^ | 0.75 | 0.274 |
| Duck | 3.24^a^ | 1.06 | 3.37^a^ | 1.07 | 0.813 |
| Capon | 4.16^c^ | 0.81 | 3.79^a^ | 0.85 | 0.091 |
| Guinea fowl | 3.40^ab^ | 1.05 | 3.89^a^ | 0.94 | 0.126 |
| Goose | 3.28^a^ | 1.09 | 3.84^a^ | 1.07 | 0.048 |
| MANOVA^2^ (F = 2.38; *P* = 0.038) | | | | |  |
| Leg meat | | | | |  |
| Broiler chicken | 3.51^ab^ | 0.64 | 3.72^a^ | 1.02 | 0.613 |
| Turkeys | 3.31^a^ | 0.93 | 3.89^a^ | 1.02 | 0.111 |
| Duck | 3.63^ab^ | 0.80 | 3.89^a^ | 1.08 | 0.416 |
| Capon | 3.76^b^ | 0.89 | 4.00^a^ | 0.84 | 0.440 |
| Guinea fowl | 3.47^ab^ | 1.03 | 3.94^a^ | 1.35 | 0.070 |
| Goose | 3.57^ab^ | 0.88 | 3.78^a^ | 1.17 | 0.561 |
| Ostrich | 3.33^a^ | 0.95 | 3.83^a^ | 1.15 | 0.065 |
| MANOVA^2^ (F = 3.80; *P* = 0.002) | | | | |  |

^a-c^ Different letters within columns indicate significant differences based on Duncan’s multiple range test at 0.05 level of significance

^1^*P*-values based on Mann–Whitney U test for comparison of means between sighted and blind panelists

^2^ Results based on MANOVA for comparison of seeing and blind panelists for all types of meat (all species)
