# Supplementary material for: Sensory evaluation of poultry meat: A comparative survey of results from normal sighted and blind people
Source: PLoS One. 2019 Jan 30;14(1):e0210722. doi: 10.1371/journal.pone.0210722 (PMC6353138; doi:10.1371/journal.pone.0210722)
Supplement: S3 Table — (DOC) [file pone.0210722.s006.doc]

**S3 Table** Data for statistical means and variability for poultry meat taste evaluation

| Type of meat | Sighted panelists | | Blind panelists | | *P*^1^ |
| --- | --- | --- | --- | --- | --- |
|  | Mean | SD | Mean | SD |  |
| Breast meat | | | | |  |
| Broiler chicken | 4.09^c^ | 0.80 | 4.05^a^ | 0.85 | 0.850 |
| Turkey | 3.31^ab^ | 0.98 | 4.05^a^ | 1.08 | 0.011 |
| Duck | 3.03^a^ | 1.24 | 3.79^a^ | 0.85 | 0.028 |
| Capon | 4.17^c^ | 0.90 | 4.32^a^ | 0.67 | 0.732 |
| Guinea fowl | 3.71^b^ | 1.01 | 3.63^a^ | 0.96 | 0.887 |
| Goose | 3.14^a^ | 1.22 | 3.68^a^ | 1.20 | 0.101 |
| MANOVA^2^ (F = 1.84; *P* = 0.103) | | | | |  |
| Leg meat | | | | |  |
| Broiler chicken | 4.29^b^ | 0.67 | 4.50^b^ | 0.79 | 0.221 |
| Turkey | 3.33^a^ | 1.03 | 4.06^ab^ | 1.00 | 0.020 |
| Duck | 3.43^a^ | 0.98 | 4.11^ab^ | 1.18 | 0.010 |
| Capon | 3.98^b^ | 1.01 | 4.17^ab^ | 1.04 | 0.432 |
| Guinea fowl | 3.41^a^ | 1.17 | 3.78^ab^ | 1.40 | 0.204 |
| Goose | 3.33^a^ | 1.07 | 4.00^ab^ | 0.97 | 0.031 |
| Ostrich | 3.41^a^ | 1.15 | 3.39^a^ | 1.33 | 0.891 |
| MANOVA^2^ (F = 1.65 *P* = 0.138) | | | | |  |

^a-c^ Different letters within columns indicate significant differences based on Duncan’s multiple range test at 0.05 level of significance

^1^*P*-values based on Mann–Whitney U test for comparison of means between sighted and blind panelists

^2^ Results based on MANOVA for comparison of seeing and blind panelists for all types of meat (all species)
