# Supplementary material for: Sensory evaluation of poultry meat: A comparative survey of results from normal sighted and blind people
Source: PLoS One. 2019 Jan 30;14(1):e0210722. doi: 10.1371/journal.pone.0210722 (PMC6353138; doi:10.1371/journal.pone.0210722)
Supplement: S4 Table — (DOC) [file pone.0210722.s007.doc]

**S4 Table** Data for statistical means and variability for poultry meat tenderness evaluation

| Type of meat | Sighted panelists | | Blind panelists | | *P*^1^ |
| --- | --- | --- | --- | --- | --- |
|  | Mean | SD | Mean | SD |  |
| Breast meat | | | | |  |
| Broiler chicken | 4.28^cd^ | 0.59 | 3.79^b^ | 0.63 | 0.017 |
| Turkey | 3.03^b^ | 0.84 | 3.11^a^ | 0.74 | 0.883 |
| Duck | 2.86^ab^ | 1.00 | 2.95^a^ | 0.85 | 0.666 |
| Capon | 4.41^d^ | 0.65 | 3.84^b^ | 1.01 | 0.039 |
| Guinea fowl | 4.00^c^ | 0.75 | 3.32^b^ | 1.00 | 0.008 |
| Goose | 2.69^a^ | 1.19 | 2.89^a^ | 1.05 | 0.412 |
| MANOVA^2^ (F = 4.11; *P =* 0.001) | | | | |  |
| Leg meat | | | | |  |
| Broiler chicken | 4.27^d^ | 0.60 | 4.39^c^ | 0.70 | 0.477 |
| Turkey | 2.96^ab^ | 0.89 | 2.94^b^ | 1.06 | 1.000 |
| Duck | 3.25^b^ | 0.82 | 3.17^b^ | 1.10 | 0.827 |
| Capon | 4.39^d^ | 0.67 | 3.56^b^ | 0.70 | <0.001 |
| Guinea fowl | 3.92^c^ | 0.72 | 3.61^b^ | 1.33 | 0.604 |
| Goose | 2.82^a^ | 0.95 | 3.44^b^ | 0.92 | 0.044 |
| Ostrich | 2.82^a^ | 0.95 | 2.39^a^ | 1.14 | 0.113 |
| MANOVA^2^ (F = 5.14; *P* < 0.001) | | | | |  |

^a-d^ Different letters within columns indicate significant differences based on Duncan’s multiple range test at 0.05 level of significance

^1^*P*-values based on Mann–Whitney U test for comparison of means between sighted and blind panelists

^2^ Results based on MANOVA for comparison of seeing and blind panelists for all types of meat (all species)
