# Supplementary material for: Sensory evaluation of poultry meat: A comparative survey of results from normal sighted and blind people
Source: PLoS One. 2019 Jan 30;14(1):e0210722. doi: 10.1371/journal.pone.0210722 (PMC6353138; doi:10.1371/journal.pone.0210722)
Supplement: S5 Table — (DOC) [file pone.0210722.s008.doc]

**S5 Table** Data for statistical means and variability for poultry meat juiciness evaluation

| Type of meat | Sighted panelists | | Blind panelists | | *P*^1^ |
| --- | --- | --- | --- | --- | --- |
|  | Mean | SD | Mean | SD |  |
| Breast meat | | | | |  |
| Broiler chicken | 4.03^cd^ | 0.90 | 3.00^ab^ | 1.05 | 0.001 |
| Turkey | 3.64^b^ | 0.91 | 2.89^ab^ | 1.45 | 0.039 |
| Duck | 2.93^a^ | 0.95 | 2.84^ab^ | 0.90 | 0.599 |
| Capon | 4.09^d^ | 1.00 | 3.47^b^ | 1.07 | 0.031 |
| Guinea fowl | 3.74^bc^ | 0.81 | 2.68^a^ | 1.11 | <0.001 |
| Goose | 2.81^a^ | 0.91 | 2.58^a^ | 0.84 | 0.348 |
| MANOVA^2^ (F = 5.88; *P* < 0.001) | | | | |  |
| Leg meat | | | | |  |
| Broiler chicken | 4.49^e^ | 0.58 | 4.22^cd^ | 0.73 | 0.224 |
| Turkey | 3.06^ab^ | 0.68 | 3.39^ab^ | 1.24 | 0.262 |
| Duck | 3.43^c^ | 0.90 | 3.67^bc^ | 1.08 | 0.444 |
| Capon | 3.96^d^ | 0.85 | 3.72^bcd^ | 0.75 | 0.265 |
| Guinea fowl | 3.86^d^ | 0.75 | 4.44^d^ | 1.04 | 0.003 |
| Goose | 3.31^bc^ | 1.01 | 3.22^ab^ | 1.00 | 0.800 |
| Ostrich | 2.82^a^ | 0.97 | 2.89^a^ | 1.32 | 0.951 |
| MANOVA^2^ (F = 2.25; *P* = 0.042) | | | | |  |

^a-e^ Different letters within columns indicate significant differences based on Duncan’s multiple range test at 0.05 level of significance

^1^*P*-values based on Mann–Whitney U test for comparison of means between sighted and blind panelists

^2^ Results based on MANOVA for comparison of seeing and blind panelists for all types of meat (all species)
