# Supplementary material for: Sensory evaluation of poultry meat: A comparative survey of results from normal sighted and blind people
Source: PLoS One. 2019 Jan 30;14(1):e0210722. doi: 10.1371/journal.pone.0210722 (PMC6353138; doi:10.1371/journal.pone.0210722)
Supplement: S6 Table — (DOC) [file pone.0210722.s009.doc]

**S6 Table** Data for statistical means and variability for poultry meat overall liking evaluation

| Type of meat | Sighted panelists | | Blind panelists | | *P*^1^ |
| --- | --- | --- | --- | --- | --- |
|  | Mean | SD | Mean | SD |  |
| Breast meat | | | | |  |
| Broiler chicken | 3.90^b^ | 0.83 | 3.58^ab^ | 1.02 | 0.242 |
| Turkey | 3.16^a^ | 0.87 | 3.74^ab^ | 1.24 | 0.086 |
| Duck | 2.93^a^ | 0.99 | 3.53^ab^ | 1.12 | 0.044 |
| Capon | 4.24^c^ | 0.80 | 4.21^b^ | 0.63 | 0.675 |
| Guinea fowl | 3.69^b^ | 0.84 | 3.26^a^ | 1.05 | 0.109 |
| Goose | 3.03^a^ | 1.03 | 3.68^ab^ | 1.11 | 0.026 |
| MANOVA^2^ (F = 3.84; *P* = 0.002) | | | | |  |
| Leg meat | | | | |  |
| Broiler chicken | 4.25^c^ | 0.66 | 4.56^c^ | 0.62 | 0.119 |
| Turkey | 3.06^a^ | 0.88 | 4.06^bc^ | 0.73 | <0.001 |
| Duck | 3.25^ab^ | 0.98 | 4.06^bc^ | 0.94 | 0.006 |
| Capon | 4.00^c^ | 0.87 | 3.78^b^ | 1.18 | 0.494 |
| Guinea fowl | 3.45^b^ | 0.99 | 3.72^b^ | 1.44 | 0.114 |
| Goose | 3.14^ab^ | 0.78 | 3.89^bc^ | 0.96 | 0.007 |
| Ostrich | 3.06^a^ | 1.03 | 3.11^a^ | 1.28 | 0.881 |
| MANOVA^2^ (F = 0.97; *P* = 0.460) | | | | |  |

^a-c^ Different letters within columns indicate significant differences based on Duncan’s multiple range test at 0.05 level of significance

^1^*P*-values based on Mann–Whitney U test for comparison of means between sighted and blind panelists

^2^ Results based on MANOVA for comparison of seeing and blind panelists for all types of meat (all species)
